# Supplementary material for: Mapping human tissues with highly multiplexed RNA in situ hybridization
Source: Nat Commun. 2024 Mar 20;15:2511. doi: 10.1038/s41467-024-46437-y (PMC10954689; doi:10.1038/s41467-024-46437-y)
Supplement: Supplementary file 1 — Supplementary Information [file 41467_2024_46437_MOESM1_ESM.pdf]

Supplementary Information

Mapping human tissues with highly multiplexed RNA in situ hybridization

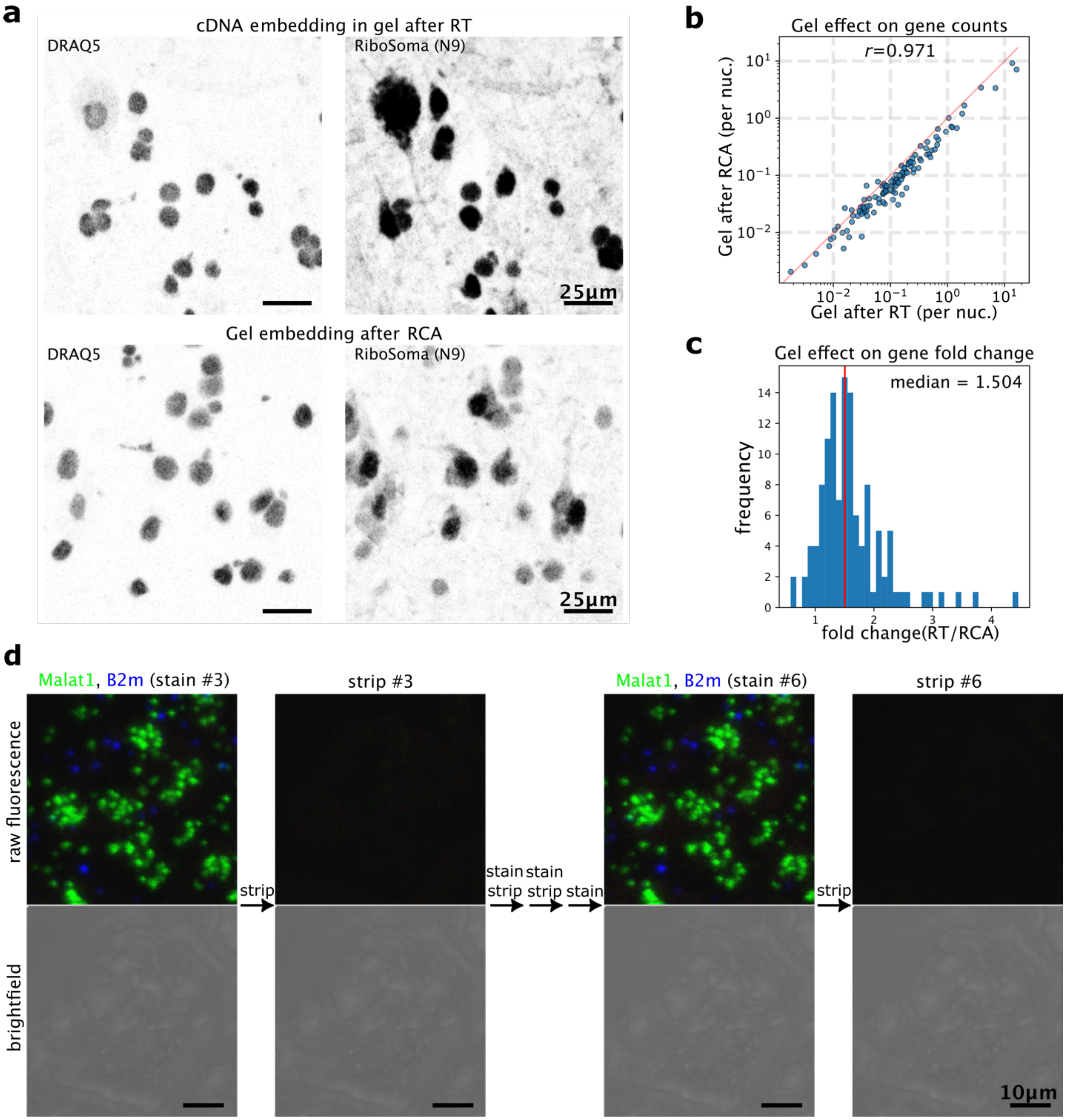

Supplementary Figure 1. Gel embedding effects and signal stability in DART-FISH. (a) Fluorescent signal from RiboSoma (randomly primed cDNA) is stronger when the cDNA is embedded in a polyacrylamide gel immediately after reverse transcription (top) compared to gel embedding after RCA (bottom). The nuclear stain (DRAQ5) shows no difference between the two conditions (left). RT: reverse-transcription, RCA: rolling circle amplification. **(b)** Scatter plot comparing the average gene count per nucleus in the two conditions in (a). Each dot represents a gene. Nuclear segmentation was chosen for counting cells to provide a fair comparison between the two conditions. **(c)** Histogram of the gene expression fold change in (b). 50% of genes show at least a 50% increase in their counts. **(d)** Rolonies are stable after multiple cycles of staining and stripping without background buildup. DART-FISH was performed on a mouse kidney tissue section with a non-combinatorial probe set containing Malat1 and B2m genes on Cy3 and Atto647N channels, respectively. Images show the rolonies of these genes on the 3rd

and 6th cycles as well as the fully cleared signal after stripping. Brightfield images serve as references for image registration. Source data are provided as a Source Data file.

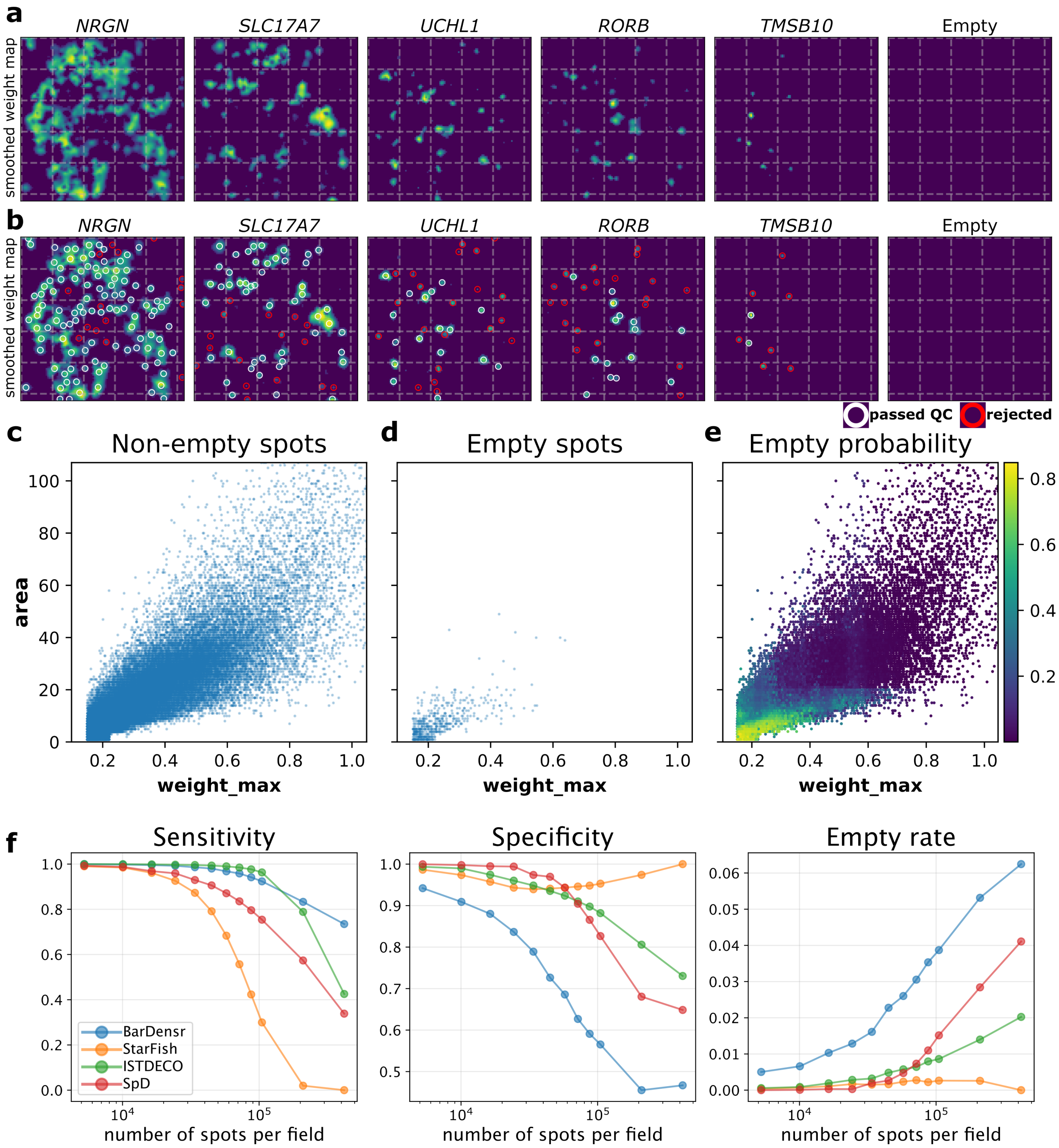

Supplementary Figure 2. SpD procedure and benchmarking. (a) Smoothed OLS maps from Fig. 1f. Lasso weight maps (Fig. 1f) undergo pixel-wise elbow filtering to select the top 1 or 2 barcodes per pixel. Unbiased weights are then obtained by fitting an ordinary linear regression (OLS) using the selected barcodes (OLS maps). OLS maps are then smoothed with a Gaussian low pass filter. **(b)** Spot detection on weight maps. For each gene, the local peaks are detected on the respective smoothed OLS map. These peaks then serve as markers for watershed

segmentation. The centroids of the segmented areas are used as spot coordinates. White and red circles are drawn around high quality and rejected spots, respectively. **(c-d)** Scatter plots of two main features extracted from segmented spots with valid barcodes representing genes (c) or empty (unused) barcodes (d). Empty barcodes tend to be smaller in area and have lower weights than valid barcodes. **(e)** Emptiness probabilities inferred from a random forest that was trained to distinguish empty from non-empty spots based on the extracted features (weight_max, weight_mean, area). A cutoff is later set on the empty probabilities to keep high quality spots. (**f**) Comparison of SpD with StarFish (naive matching), BarDensr and ISTDECO (deconvolution-based methods) on synthetic images with varying degrees of difficulty. Naive matching is only sensitive when the data is sparse. Deconvolution-based methods perform more robustly against dense data.

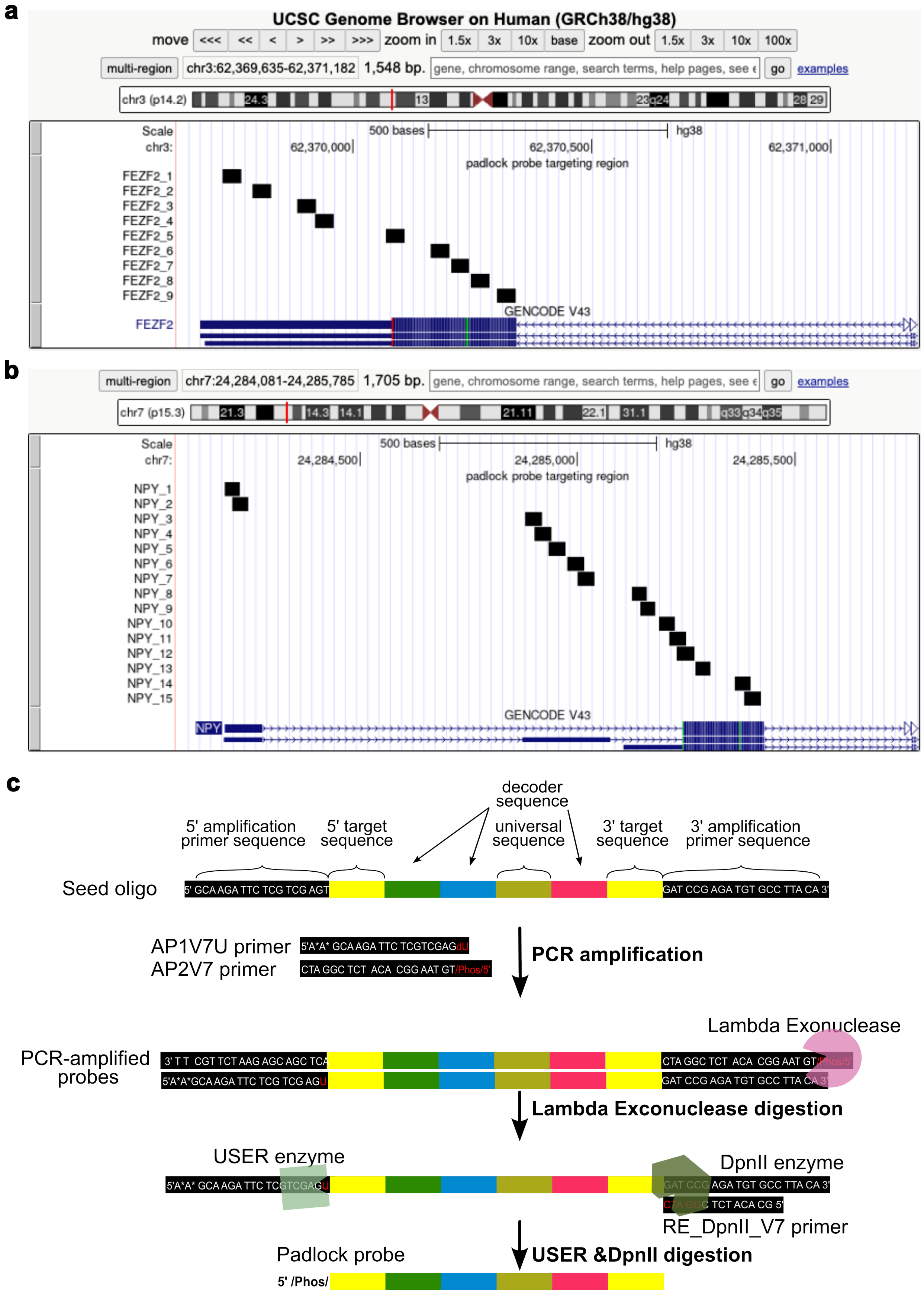
a
UCSC Genome Browser on Human (GRCh38/hg38)
move <<< << < > >> >>> zoom in 1.5x 3x 10x base zoom out 1.5x 3x 10x 100x
multi-region chr3:62,369,635-62,371,182 1,548 bp. gene, chromosome range, search terms, help pages, see e go examples
chr3 (p14.2)
Scale
chr3:
500 bases
hg38
62,370,000
62,370,500
62,371,000
padlock probe targeting region
FEZF2_1
FEZF2_2
FEZF2_3
FEZF2_4
FEZF2_5
FEZF2_6
FEZF2_7
FEZF2_8
FEZF2_9
GENCODE V43
FEZF2
b
multi-region chr7:24,284,081-24,285,785 1,705 bp. gene, chromosome range, search terms, help pages, see e go examples
chr7 (p15.3)
Scale
chr7:
500 bases
hg38
24,284,500
24,285,000
24,285,500
padlock probe targeting region
NPY_1
NPY_2
NPY_3
NPY_4
NPY_5
NPY_6
NPY_7
NPY_8
NPY_9
NPY_10
NPY_11
NPY_12
NPY_13
NPY_14
NPY_15
GENCODE V43
NPY
c
decoder sequence
5' amplification primer sequence
5' target sequence
universal sequence
3' target sequence
3' amplification primer sequence
Seed oligo
5' GCA AGA TTC TCG TCG AGT
GAT CCG AGA TGT GCC TTA CA 3'
AP1V7U primer
5'A*A* GCA AGA TTC TCGTCGAGdU
AP2V7 primer
CTA GGC TCT ACA CGG AAT GT/Phos/5'
PCR amplification
Lambda Exonuclease
PCR-amplified probes
3' T T CGT TCT AAG AGC AGC TCA
CTA GGC TCT ACA CGG AAT GT/Phos/5'
5'A*A*GCA AGA TTC TCG TCG AGU
GAT CCG AGA TGT GCC TTA CA 3'
Lambda Exconuclease digestion
USER enzyme
DpnII enzyme
5'A*A* GCA AGA TTC TCGTCGAGU
GAT CCG AGA TGT GCC TTA CA 3'
CTA GGC TCT ACA CG 5'
RE_DpnII_V7 primer
Padlock probe
USER &DpnII digestion
5' /Phos/

Supplementary Figure 3. Probe design and production strategy. (a) A genome browser view showing the target locations for individual padlock probes. Up to 50 padlock probes are designed to tile the constitutive exons. **(b)** To obtain more probes targeting short genes, we allow overlapping target sequences for padlock probes (*NPY* as an example with 893 nucleotides) **(c)** Enzymatic production of padlock probes from an oligo pool. A probe set is PCR amplified with a pair of probe-set specific amplification primers from an oligo pool. The forward primer carries a 5’ phosphorothioate modification to prevent exonuclease digestion and a 3’ deoxyuridine modification, while the reverse primer carries a 5’ phosphorylation modification to promote digestion by lambda exonuclease. The 5’ and 3’ ends of the amplicons are cleaved by USER and DpnII, respectively, to obtain single-stranded padlock probes with a 5’ phosphate.

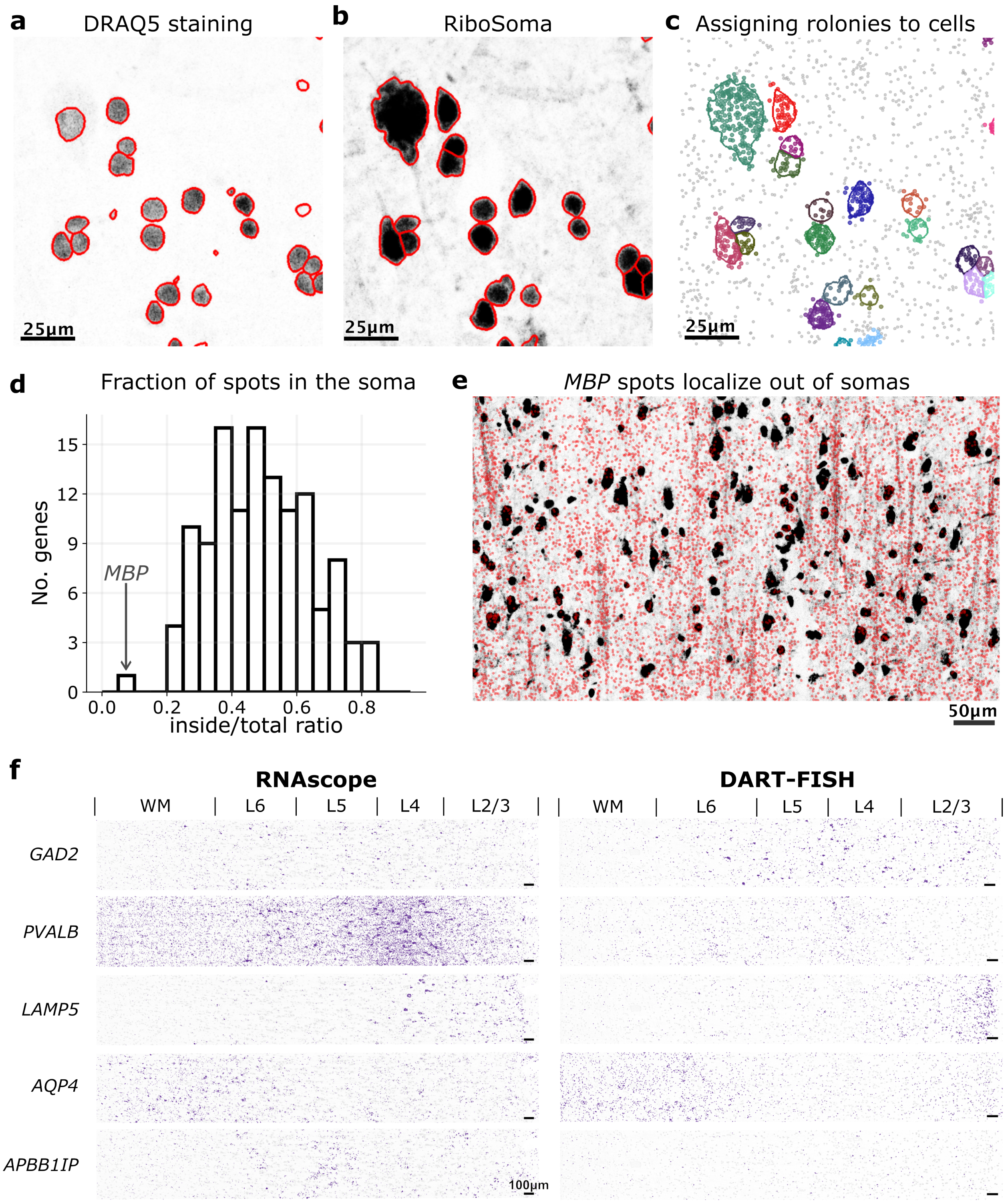

Supplementary Figure 4. RiboSoma, cell segmentation and RNAscope validation. (a) Segmentation of cells using only nuclear (DRAQ5) or **(b)** both DRAQ5 and RiboSoma (randomly primed) staining. RiboSoma resolves cell bodies more confidently. **(c)** Assigning decoded rolonies to the closest segmented cell. Transcripts that are too far from cell boundaries are discarded. **(d)** Histogram showing the fraction of spots inside the segmented cells for each gene. *MBP* encoding Myelin basic protein has the lowest fraction of spots inside the cells. **(e)** An

example of *MBP* being expressed outside the soma. Every red dot is a decoded *MBP* transcript on the background of RiboSoma (contrast is increased for clarity). *MBP* spots seem to co-localize with the RiboSoma signal over long threads that resemble axons. **(f)** RNAscope validation of DART-FISH in the human M1C with genes *GAD2*, *PVALB*, *LAMP5*, *AQP4*, *APBB1IP* on parallel sections. Source data are provided as a Source Data file.

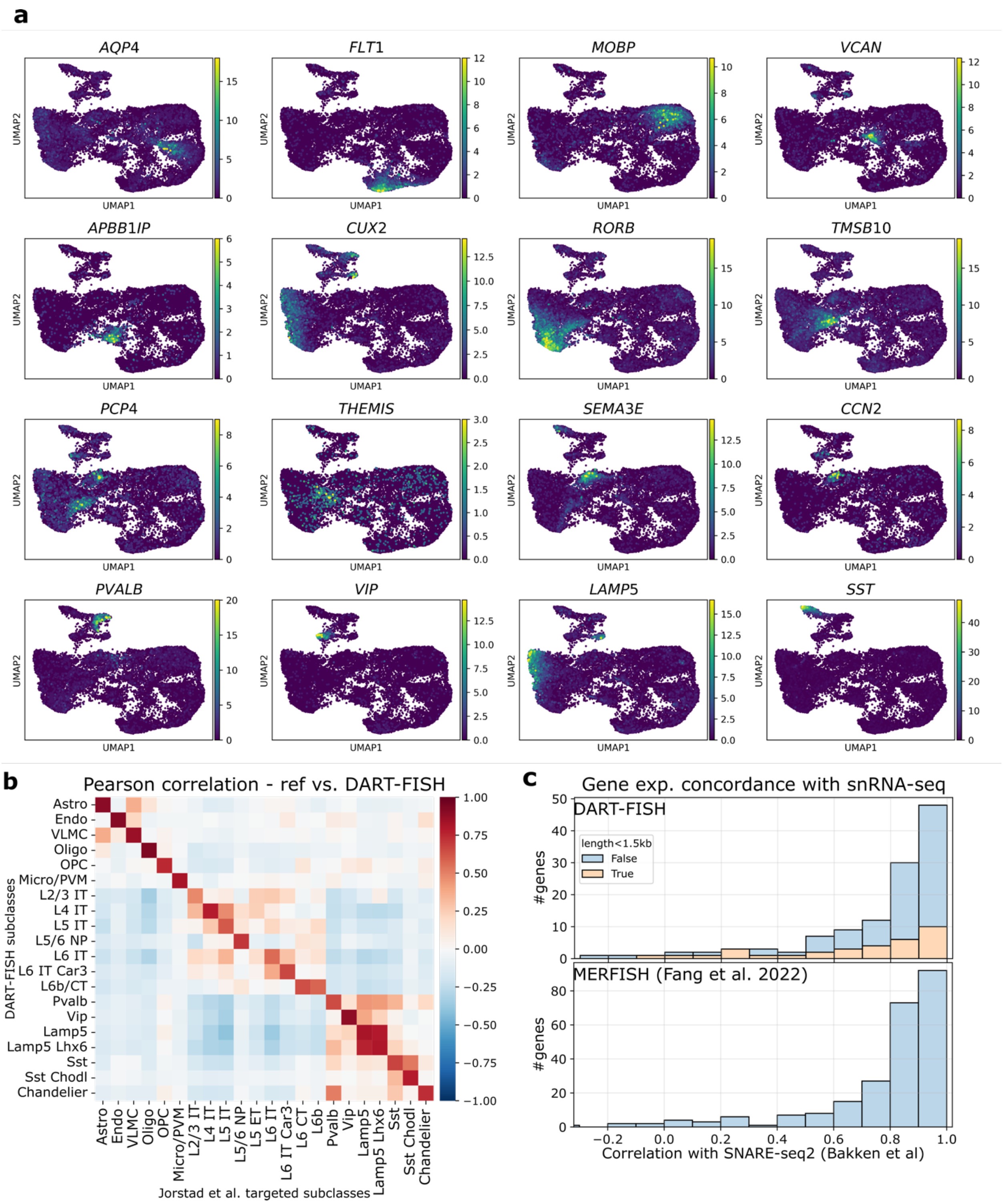

Supplementary Figure 5. Cell annotation and expression of marker genes in human M1C. **(a)** UMAP embedding of the DART-FISH M1C data colored by transcripts counts for 16 cell type markers. **(b)** Pearson's correlation of DART-FISH subclasses with the snRNA-seq reference subclasses used for annotation[1] **(c)** Histogram of concordance values for genes in DART-FISH (top) and MERFISH (bottom, sample H18.06.006.MTG.250.expand.rep1[2]). Concordance is defined as the Pearson's correlation of expression levels across subclasses between SNARE-

seq2[3] and the spatial assay. The histogram for DART-FISH is color coded to show the performance of short genes (constitutive exon length <1.5kb). Source data are provided as a Source Data file.

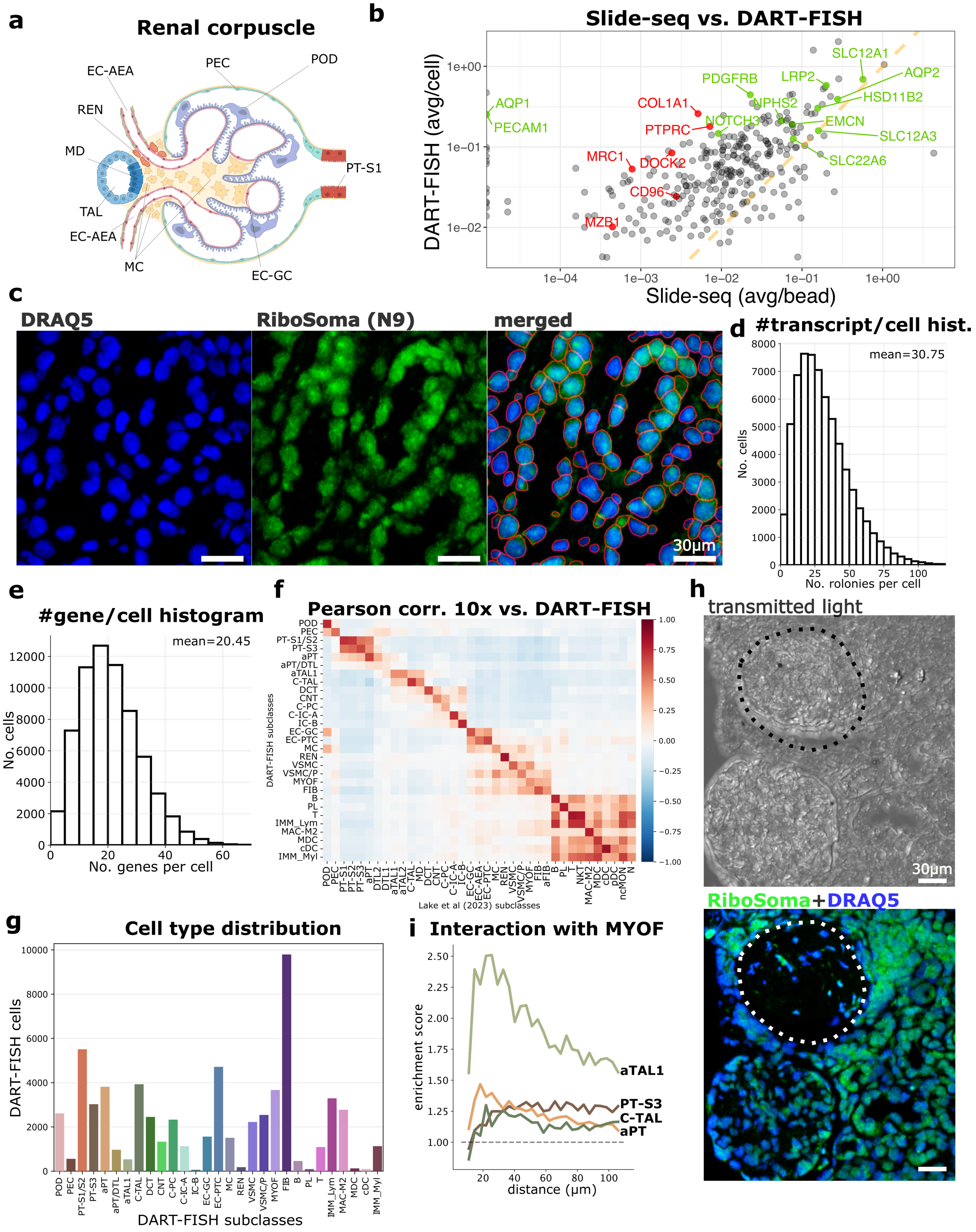

Supplementary Figure 6. Cell types and gene expression in the human kidney. (a) Diagram of the cell types composing the renal corpuscle and the juxtaglomerular apparatus[4]. **(b)**

Scatter plot comparing average gene counts per bead in Slide-seq (Puck_200903_06 from a healthy patient[5]) with average counts per cell in DART-FISH (r=0.609). Green dots represent canonical cell type markers while red dots are immune markers, suggesting high inflammation in the DART-FISH samples. The orange line indicates equal average counts across the two technologies. The top 150 highly expressed genes in slide-seq had on median 2.2x lower average transcripts per bead than average transcripts per cell in DART-FISH. **(c)** RiboSoma (randomly primed cDNA, middle) resolves tubular morphology better than the nuclear stain (left) and enhances cell segmentation (right). **(d)** Histogram of the number of rolonies per cell in >65,000 cells. There are on average ~30 decoded transcripts per cell. **(e)** Histogram of the number of detected genes per cell in the kidney, averaging at 20 unique genes per cell. **(f)** Pearson's correlation of average DART-FISH subclasses with the average snRNA-seq reference subclasses used for annotation[5] **(g)** Bar plot showing the number of cells from each annotated subclass in the human kidney from n=1 section. High numbers of immune cells and fibroblasts are suggestive of inflammation and fibrosis. **(h)** Transmitted light (top) and RiboSoma overlaid with nuclear stain (bottom) of the ROI shown in Fig. 4g. The cells in the sclerosed glomerulus (dashed line) are mostly replaced by scar tissue as shown by the occupied space in the transmitted light view. **(i)** Plot showing the co-occurrence enrichment[6] of some cell types with MYOF at a range of distances, suggesting an interaction between MYOF with aTAL1 cells whereas there is no apparent co-occurrence enrichment between MYOF and aPT, or healthy PT-S3 and C-TAL. Source data are provided as a Source Data file.

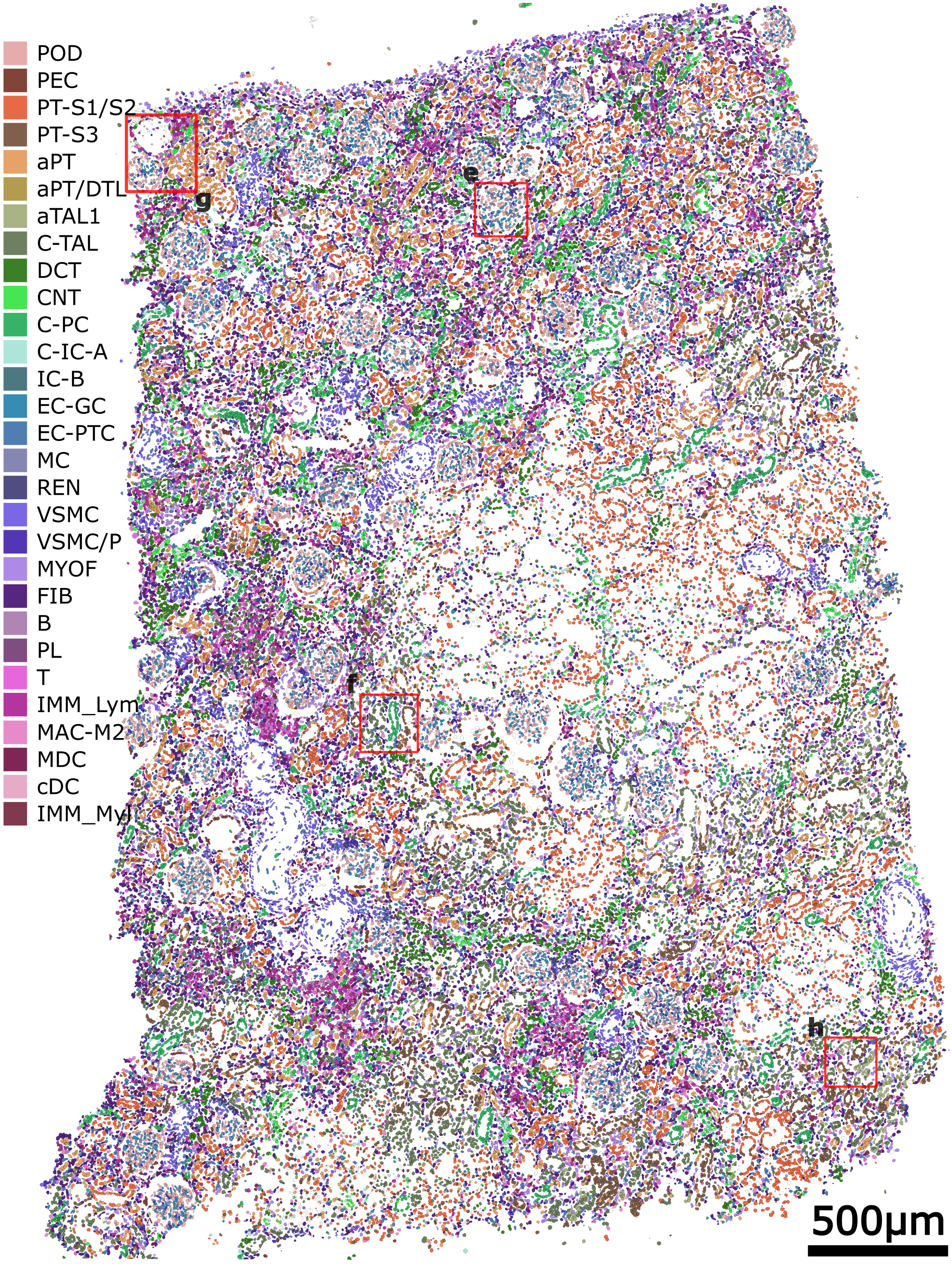
POD
PEC
PT-S1/S2
PT-S3
aPT
aPT/DTL
aTAL1
C-TAL
DCT
CNT
C-PC
C-IC-A
IC-B
EC-GC
EC-PTC
MC
REN
VSMC
VSMC/P
MYOF
FIB
B
PL
T
IMM_Lym
MAC-M2
MDC
cDC
IMM_Myl
g
e
f
h
500μm

Supplementary Figure 7. Spatial mapping of cell types in the human kidney. A whole tissue rendering of all segmented cells colored by all annotated subclasses. Red boxes correspond to ROIs in Fig. 4e-h

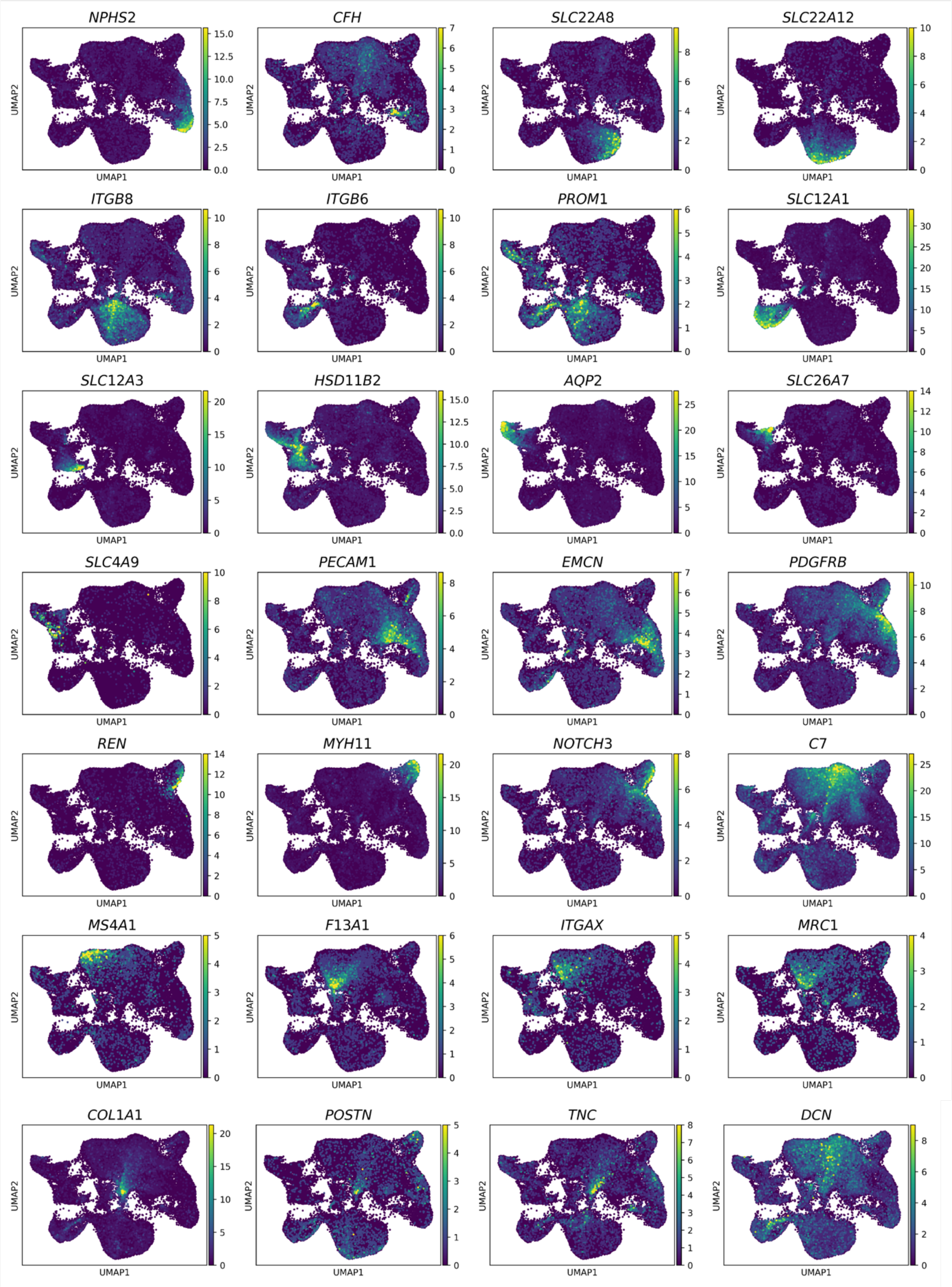
NPHS2
CFH
SLC22A8
SLC22A12
ITGB8
ITGB6
PROM1
SLC12A1
SLC12A3
HSD11B2
AQP2
SLC26A7
SLC4A9
PECAM1
EMCN
PDGFRB
REN
MYH11
NOTCH3
C7
MS4A1
F13A1
ITGAX
MRC1
COL1A1
POSTN
TNC
DCN
UMAP1
UMAP2

Supplementary Figure 8. Expression of marker genes in the human kidney. UMAP embedding of the DART-FISH data on human kidney colored by different marker genes in Fig. 4e.

Supplementary Table 1. The glossary for the abbreviated cell types and the technical terms.

Abbreviation	full name
General terms	
DART-FISH	decoding amplified targeted transcripts with fluorescence in situ hybridization
FOV	field of view
ROI	region of interest
RCA	rolling circle amplification
smFISH	single-molecule fluorescence in situ hybridization
SNR	signal-to-noise ratio
NA	numerical aperture
snRNA-seq	single-nucleus RNA-seq
SNARE-seq	single-nucleus chromatin accessibility and mRNA expression sequencing
Brain terminology	
M1C	primary motor cortex
IT	intratelencephalic
NP	near-projecting
CT	corticothalamic
Astro	astrocyte
endo	endothelial cell
VLMC	vascular and leptomeningeal cell
Oligo	oligodendrocyte
OPC	oligodendrocyte precursor cell
Micro/PVM	microglia/perivascular macrophage
WM	white matter
Kidney terminology	
POD	podocyte
PEC	parietal epithelial cell
PT	proximal tubule
aPT	adaptive PT
PT-S1/S2	S1 or S2 segments of proximal tubules
PT-S3	S3 segment of proximal tubules
DTL	descending thin limb
C-TAL	cortical thick ascending limb
aTAL	adaptive TAL
DCT	distal convoluted tubule
CNT	connecting tubules
C-IC-A	cortical intercalated cell type A
IC-B	intercalated cell type B
C-PC	cortical principal cells of the collecting duct
EC-GC	glomerular capillary endothelial cell
EC-PTC	peritubular capillary endothelial cell
VSCM	vascular smooth muscle cell
MC	mesangial cell
REN	renin-positive juxtaglomerular granular cell
VSMC/P	vascular smooth muscle cell/pericyte
FIB	fibroblast
MYOF	myofibroblast
MAC-M2	M2 macrophage
IMM-Lym	lymphoid cell
IMM-Myl	myeloid cell
T, B, PL	T cell, B cell, Plasma cell
MDC	Monocyte-derived cell
cDC	Classical dendritic cell
H&E	Hematoxylin and Eosin

Supplementary Table 2. Comparing DART-FISH with similar methods.

Technology	#genes	#decoding cycles	barcode space used[a]	enzymatic signal amp.	Objective magnification[b]	#probes per gene	Probe set cost for 300 genes[d]	between cycle preparation time	cell segmentation	tissues tested
DART-FISH	120-300	7	22-31%	Yes	20x	50	$5200 (Twist Bio)	45 minutes	RiboSoma, nuclear	Human brain, Human Kidney
ISS[7]	99	5	15%[c]	Yes	20x	7-8	$136,000 (IDT 4nm Ultramer plate with 5' phosphorylation)	>1.5 hours	nuclear	Mouse Brain
HybISS[8]	120	5	11%[c]	Yes	40x	5	$52,000 (IDT 4nm Ultramer plate without 5' phosphorylation)	3.5 hours	nuclear	Human brain, Mouse brain
STARmap[9]	160-1000	6	15-97%[c]	Yes	40x	4	$38,000 (IDT plates with 5' phosphorylation)	4.5 hours	nuclear	Mouse brain
MERFISH[2]	250-4000	16	6-96%	No	60x	60	$5200 (Twist Bio)	35 minutes	Poly dT, nuclear	Human brain, Mouse brain

[a] Number of used barcodes over the number of valid barcodes. Lower ratios enable better error detection and signal demixing.
[b] Higher magnification is required for smaller and dimmer features. On the other hand, lower magnification objectives can image larger areas faster.
[c] 4-color imaging was used instead of 3-color imaging
[d] Cost of probe sets cannot be accurately compared across technologies for the following reasons: 1) Each technology uses a different number of probes per gene. For example, smFISH-based technologies (e.g., MERFISH) need a minimum number of probes for the signal to be detectable, while padlock-probe based technologies enjoy higher sensitivity with more padlocks per gene. Cost of direct synthesis scales linearly with the number of probes, thus technologies that use direct synthesis tend to keep the number of probes per gene low 3) each technology may use a different mass of probes per experiment which makes it very difficult to estimate cost per experiment. We tried to estimate the cost of purchasing the probe sets with the best knowledge obtained from the cited manuscripts. These values do not include the costs associated with the preparation of these probes for an experiment (e.g., amplification, phosphorylation)

Supplementary References

1. Jorstad, N. L. *et al.* Transcriptomic cytoarchitecture reveals principles of human neocortex organization. *Science* **382**, eadf6812 (2023).
2. Fang, R. *et al.* Conservation and divergence of cortical cell organization in human and mouse revealed by MERFISH. *Science* **377**, 56–62 (2022).
3. Bakken, T. E. *et al.* Comparative cellular analysis of motor cortex in human, marmoset and mouse. *Nature* **598**, 111–119 (2021).
4. Kidney Precision Medicine Project. Schemata of the human nephron and renal corpuscle developed by the Kidney Precision Medicine Project. (2021) doi:10.48698/DEM4-0Q93.
5. Lake, B. B. *et al.* An atlas of healthy and injured cell states and niches in the human kidney. *Nature* **619**, 585–594 (2023).
6. Palla, G. *et al.* Squidpy: a scalable framework for spatial omics analysis. *Nat. Methods* **19**, 171–178 (2022).
7. Qian, X. *et al.* Probabilistic cell typing enables fine mapping of closely related cell types in situ. *Nat. Methods* **17**, 101–106 (2020).
8. Gyllborg, D. *et al.* Hybridization-based in situ sequencing (HybISS) for spatially resolved transcriptomics in human and mouse brain tissue. *Nucleic Acids Res.* **48**, e112 (2020).
9. Wang, X. *et al.* Three-dimensional intact-tissue sequencing of single-cell transcriptional states. *Science* **361**, (2018).
