# Supplementary material for: Mapping human tissues with highly multiplexed RNA in situ hybridization
Source: Nat Commun. 2024 Mar 20;15:2511. doi: 10.1038/s41467-024-46437-y (PMC10954689; doi:10.1038/s41467-024-46437-y)
Supplement: Supplementary file 4 — Description of Additional Supplementary Files [file 41467_2024_46437_MOESM4_ESM.pdf]

Description of Additional Supplementary Files

File Name: Supplementary Data 1

Description: Decoding probes, probe production and DART-FISH primers, reagents

File Name: Supplementary Data 2

Description: Brain padlock probe sequences, gene lengths and concordances

File Name: Supplementary Data 3

Description: Kidney padlock probe sequences
